# Virtual elastography ultrasound via generative adversarial network for breast cancer diagnosis

**DOI:** 10.1038/s41467-023-36102-1

**Published:** 2023-02-11

**Authors:** Zhao Yao, Ting Luo, YiJie Dong, XiaoHong Jia, YinHui Deng, GuoQing Wu, Ying Zhu, JingWen Zhang, Juan Liu, LiChun Yang, XiaoMao Luo, ZhiYao Li, YanJun Xu, Bin Hu, YunXia Huang, Cai Chang, JinFeng Xu, Hui Luo, FaJin Dong, XiaoNa Xia, ChengRong Wu, WenJia Hu, Gang Wu, QiaoYing Li, Qin Chen, WanYue Deng, QiongChao Jiang, YongLin Mou, HuanNan Yan, XiaoJing Xu, HongJu Yan, Ping Zhou, Yang Shao, LiGang Cui, Ping He, LinXue Qian, JinPing Liu, LiYing Shi, YaNan Zhao, YongYuan Xu, WeiWei Zhan, YuanYuan Wang, JinHua Yu, JianQiao Zhou

**Affiliations:** 1 School of Information Science and Technology, Fudan University, Shanghai, China; 2 Department of Ultrasound, Ruijin Hospital, Shanghai Jiaotong University School of Medicine, Shanghai, 200025 China; 3 Department of Medical Ultrasound, Yunnan Cancer Hospital & The Third Affiliated Hospital of Kunming Medical University, Kunming, 650118 China; 4 Department of Ultrasound in Medicine, Shanghai Jiao Tong University Affiliated Sixth People’s Hospital, Shanghai Institute of Ultrasound in Medicine, Shanghai, 200233 China; 5 Department of Ultrasound, Minhang Hospital, Fudan University, Shanghai, 201199 China; 6 Department of Ultrasonography, Fudan University Shanghai Cancer Center, Shanghai Medical College, Fudan University, Shanghai, 200032 China; 7 Department of Ultrasound, Shenzhen People’s Hospital, Guangzhou, Guangdong China; 8 Department of Ultrasound Medicine, The First Affiliated Hospital of Xi’an Jiaotong University, Xi’an, 710061 China; 9 Department of Ultrasound, People’s Hospital of Henan Province, Zhengzhou, 450000 China; 10 Department of Ultrasound Diseases, Tangdu Hospital, Four Military Medical University, Xi’an, 710038 China; 11 Department of Ultrasound, Sichuan Provincial People’s Hospital, University of Electronic Science and Technology of China, Chengdu, 610072 China; 12 Department of Ultrasound, Sun Yat-sen Memorial Hospital, Sun Yat-sen University, Guangzhou, 510120 China; 13 Department of Ultrasound, General Hospital of Northern Theater Command, 110000 Shenyang, China; 14 Department of Ultrasound, Affiliated Hangzhou First people’s Hospital, Zhejiang University School of Medicine, Hangzhou, 310006 China; 15 Department of Ultrasound, The Third Xiangya Hospital of Central South University, Changsha, 410013 China; 16 Department of Ultrasound, Peking University Third Hospital, Beijing, 100191 China; 17 Department of Ultrasound, Beijing Friendship Hospital, Capital Medical University, Beijing, 100050 China; 18 Department of Ultrasound, Affiliated Hospital of Guizhou Medical University, Guiyang, Guizhou 550004 China; 19 Department of Ultrasound, Second Affiliated Hospital of Zhejiang University, School of Medicine, Hangzhou, 310009 China

**Keywords:** Machine learning, Breast cancer, Data mining

## Abstract

Elastography ultrasound (EUS) imaging is a vital ultrasound imaging modality. The current use of EUS faces many challenges, such as vulnerability to subjective manipulation, echo signal attenuation, and unknown risks of elastic pressure in certain delicate tissues. The hardware requirement of EUS also hinders the trend of miniaturization of ultrasound equipment. Here we show a cost-efficient solution by designing a deep neural network to synthesize virtual EUS (V-EUS) from conventional B-mode images. A total of 4580 breast tumor cases were collected from 15 medical centers, including a main cohort with 2501 cases for model establishment, an external dataset with 1730 cases and a portable dataset with 349 cases for testing. In the task of differentiating benign and malignant breast tumors, there is no significant difference between V-EUS and real EUS on high-end ultrasound, while the diagnostic performance of pocket-sized ultrasound can be improved by about 5% after V-EUS is equipped.

## Abstract (web summary)

The current use of elastography ultrasound faces challenges, including vulnerability to subjective manipulation, echo signal attenuation, unknown risks of elastic pressure and high imaging hardware cost. Here, the author shows a virtual elastography to empower low-end ultrasound devices with state-of-art elastography function.

## Introduction

Ultrasound imaging (US) is an essential component of modern medical imaging technology. Elastography ultrasound imaging (EUS), as a widely used ultrasound imaging modality, can be used to assess the biomechanical properties of soft tissues. EUS provides distinctive information different from other US modalities and plays an increasingly important role in diagnosing many diseases, especially tumors, with significant clinical value^1,2^.

With the rapid development of integrated circuits, an important trend in US equipment is towards miniaturization and portability to take full advantage of real-time, non-invasive, inexpensive, and easily accessible US^3,4^. Due to the hardware requirements of EUS, none of the existing pocket-sized ultrasound instruments are able to provide elastography modality, which has become an obstacle to the widespread use of miniaturized ultrasound equipment^3,5^. On the other hand, compared with B-mode ultrasound imaging (BUS), EUS is more susceptible to subjective manipulation, including probe position, applied pressure, and frequency of compression, which dictates a higher operator dependence and longer learning curve^6^. In addition, EUS requires the calculation of tissue displacement based on ultrasound echo signals, and the accuracy of displacement calculation is strongly influenced by signal attenuation, with the consequence that the quality of EUS of deep tissues degrades significantly. Furthermore, as EUS relies on stress changes to capture the elasticity of tissue, and the biomechanical properties of delicate tissues, such as carotid plaque, eye, and brain tissue, are not well understood, leading to no clear conclusions about the safety of EUS in the diagnosis of these lesions.

With the rapid development of artificial intelligence, deep learning-based medical image synthesis technology offers promising solutions to many data-driven clinical application challenges. For example, data synthesis technology can improve the imaging quality of low-end acquisition equipment and break through the limits of the original imaging methods in various aspects such as imaging speed^7^, resolution^8^, modality^9^, and slice staining techniques^10^.

**Fig. 1:**
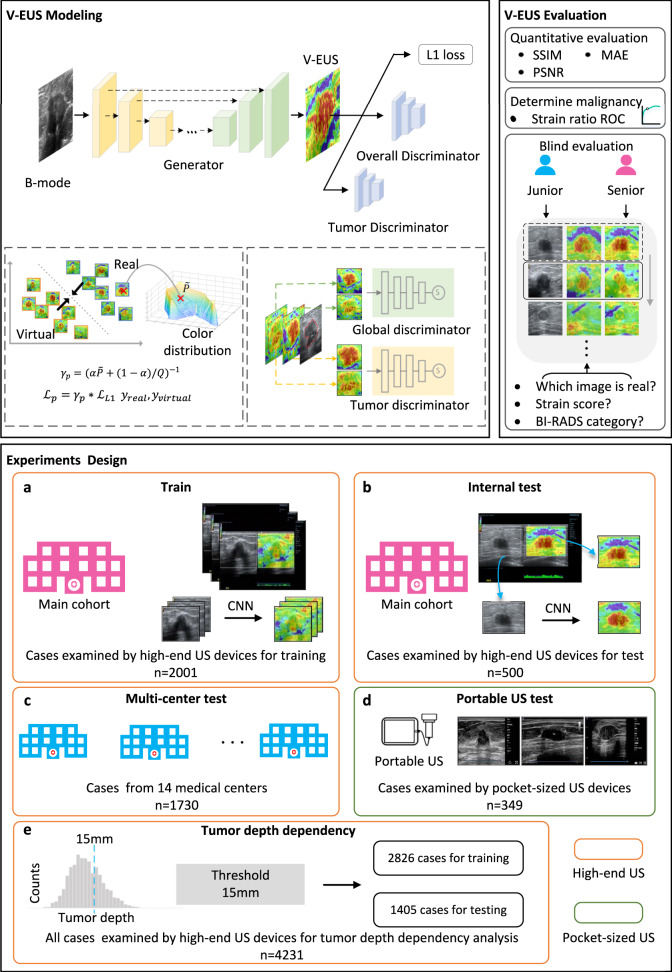
Overview of the experimental workflow. In V-EUS modeling, the generator takes BUS as input and synthesizes V-EUS, and then the discriminator determines whether the input EUS is real. A color rebalance module and a tumor discrimination module are designed to regularize the model. In V-EUS evaluation, the performance of the model is inspected from three aspects: image quality metrics, determining tumor malignancy, and blind evaluation. We design five experiments in this work. **a** The model is trained on 2001 high-quality US images from the main cohort. **b** The hold-out 500 high-quality US images are used as an internal test. **c** We evaluate the trained model on an external multi-center test cohort with 1730 high-quality US images. **d** The model is further evaluated on a more challenging dataset containing 349 low-quality US images collected from pocket-sized US devices. **e** In order to analyze tumor depth dependency, all high-quality US images are divided according to different tumor depth intervals, modeling with 15 mm as division thresholds.

To tackle the barriers mentioned above to use EUS in clinical applications, in this paper, we propose a cost-efficient solution by designing an image synthesis method based on deep learning. Specifically, a virtual EUS (V-EUS) reconstruction method based on generative adversarial network (GAN) is proposed to establish an end-to-end mapping relationship from BUS to EUS. To fully validate the clinical value of V-EUS, we choose the clinical problem of breast cancer diagnosis and validate it in 4580 breast tumor cases from 15 medical centers. In order to obtain an accurate elasticity assessment of the tumor region and to make the color distribution of V-EUS highly compatible with the one of real EUS, we propose to integrate a tumor discriminator module and a color balancing module in the GAN framework. We perform extensive evaluations of V-EUS, which are carried out in terms of the following data organization: internal validation on 2501 cases, external validation on 14 cohorts of 1730 cases from 14 centers, and another external validation on 349 cases obtained from pocket-sized ultrasound equipment. The comparisons between V-EUS and real EUS are performed from the following perspectives: numerical indicators of image similarity, visual evaluation by radiologists with different years of experience, contribution to the diagnostic accuracy of breast cancer, stability of elastography of different imaging depth, diagnostic effectiveness of pocket-sized ultrasound instrument with or without V-EUS. The overview of the construction and evaluation of V-EUS is shown in Fig. 1.

## Results

### Patient and breast lesion Characteristics

All radiologists involved in the project at each sub-center had at least 3 years of experience in breast EUS and were uniformly trained in imaging methods prior to the start of the study. The acquired imaging data were stored on hard disks and sent to the study center for analysis. The mean age of 4580 cases was 48 ± 14 age, including 4578 women and 2 men. These included 2226 malignant tumors and 2354 benign tumors, with the most common of the malignant tumors being invasive ductal carcinoma and the most common of the benign tumors being fibroadenoma. The patient demographics and breast lesion characteristics are listed in Table 1.

**Table 1:** patient demographics and breast lesion characteristics

Characteristics	Main cohort	External cohort	Portable US
Total no. of cases	2501	1730	349
Age,years: median (25th, 75th, percentiles)	49 (39, 60)	44 (24, 52)	48 (39, 61)
**Lesion size (mm)**			
<10 (%)	545 (21.8%)	259 (15.0%)	41 (11.7%)
10~19.9 (%)	1100 (44.0%)	863 (49.9%)	156 (44.7%)
20~30 (%)	546 (21.8%)	450 (26.0%)	108 (30.9%)
>30 (%)	310 (12.4%)	158 (9.1%)	44 (12.6%)
**Lesion depth (mm)**			
<10 (%)	385 (15.4%)	287 (16.6%)	72 (20.6%)
10~14.9 (%)	1256 (50.2%)	893 (51.6%)	184 (52.7%)
15~20 (%)	710 (28.4%)	461 (26.6%)	80 (22.9%)
>20 (%)	150 (6.0%)	89 (5.1%)	13 (3.7%)
**BI-RADS category**^a^			
2 (%)	5 (0.2%)	1 (0.1%)	2 (0.6%)
3 (%)	335 (13.4%)	595 (34.4%)	39 (11.2%)
4a (%)	728 (29.1%)	443 (25.6%)	110 (31.5%)
4b (%)	415 (16.6%)	245 (14.2%)	47 (13.5%)
4c (%)	643 (25.7%)	296 (17.1%)	92 (26.4%)
5 (%)	375 (15.0%)	150 (8.7%)	59 (16.9%)
**Lesion location**			
Upper outer (%)	1050 (42.0%)	780 (45.1%)	154 (44.2%)
Upper inner (%)	710 (28.4%)	438 (25.3%)	92 (26.3%)
Lower inner (%)	215 (8.6%)	167 (9.7%)	32 (9.2%)
Lower outer (%)	526 (21.0%)	345 (19.9%)	71 (20.3%)
**Lesion type**			
Invasive carcinoma^b^ (%)	1100 (44.0%)	587 (33.9%)	174 (49.9%)
Carcinoma in situ (%)	170 (6.8%)	28 (1.6%)	16 (4.6%)
Other malignant^c^ (%)	80 (3.2%)	65 (3.8%)	6 (1.7%)
Fibroadenoma (%)	730 (29.2%)	700 (40.5%)	90 (25.8%)
Other benign^d^ (%)	421 (16.8%)	350 (20.2%)	63 (18.0%)

^a^The BI-RADS category summarized in this table is based on the interpretation of the US radiologists who originally performed the US examinations.

^b^The invasive carcinoma includes invasive ductal carcinoma, invasive lobular carcinoma.

^c^The other malignant includes mucinous carcinoma and non-specific malignant results.

^d^Includes adenosis, hyperplasia, papillomas tumours and benign phyllodes.

### Quantitative evaluation metrics and subjective evaluation methods of V-EUS

In order to assess the quality of V-EUS comprehensively, we perform both quantitative and subjective evaluations. Quantitative evaluations are performed in following two aspects: similarity between V-EUS and real EUS and the efficacy of V-EUS in the diagnosis of breast cancer. We use structure similarity index measurement (SSIM), mean absolute percentage error (MAPE), and color histogram correlation (CHC) to quantitatively measure the reconstruction error between V-EUS and real EUS. These three indexes quantitatively compare V-EUS with EUS in terms of similarity of image structure, similarity of elasticity values, and similarity of color distribution, respectively. As an intuitive interpretation, large SSIM and CHC values indicate good agreement between V-EUS and real EUS, while large MAPE values indicate large synthetic errors. The calculation methods of these three indexes are detailed in Methods. We further quantify the stiffness of the tumor by calculating the strain ratio (SR), which is a simi-quantitative assessment method and defined as the ratio of the deformation of the normal breast tissue to the tumor tissue, and then analyze its diagnostic efficacy by using the receiver operating characteristic (ROC) curve. The detailed calculation process of SR is illustrated in Methods.

In addition to the objective evaluation, we also conduct subjective blind evaluations on V-EUS. Both junior and senior US radiologists are required to perform visual Turing tests to evaluate the visual fidelity of V-EUS. The procedure of subjective evaluations is described in Methods.

### V-EUS evaluation in the internal validation set

**Fig. 2:**
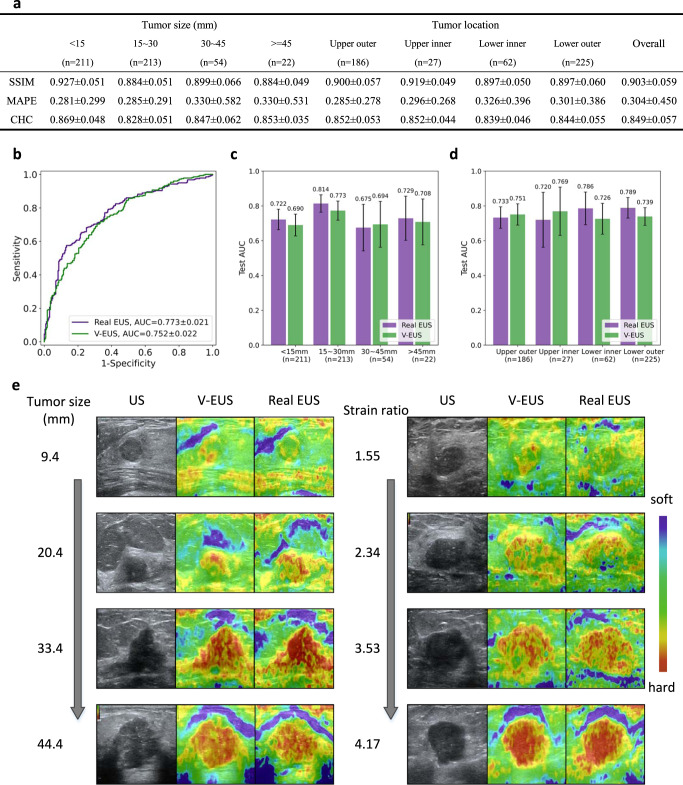
Performance of the deep learning model on the internal validation set. **a** Detailed quantitative metrics comparison stratified by tumor size and tumor location. **b** Comparison of ROCs between real EUS and V-EUS in determining breast tumor malignancy. **c** Comparison of diagnostic performance stratified by tumor size. n indicates the number of cases in the interval. Error bar indicates 95% confidence intervals of AUC. **d** Comparison of diagnostic performance stratified by tumor location. *n* indicates the number of cases in the interval. Error bar indicates 95% confidence intervals of AUC. **e** Results of several examples. Source data are provided as a Source Data file.

The overall values of SSIM, MAPE and CHC are 0.903, 0.304 and 0.849, respectively, which indicates a good agreement between V-EUS and real EUS. The detailed quantitative metrics stratified by tumor size and tumor location is shown in Fig. 2a.

An essential aspect of evaluating V-EUS is the application in the clinical practice, differentiating between benign and malignant breast tumors in our application. We calculate the SR values of real EUS and V-EUS, respectively, and use the SR values to calculate the AUCs for breast cancer diagnosis. The performance of SR values obtained from real EUS is similar to that of V-EUS, with AUC of 0.773 and 0.752, respectively (*p* = 0.396, Fig. 2b). In the task of breast cancer diagnosis, we usually choose a smaller diagnostic threshold to ensure high sensitivity, and it can be seen from the ROC that the diagnostic performance of real EUS and V-EUS is similar at this time. Further, we compared the diagnostic performance of V-EUS and real EUS stratified by tumor size (Fig. 2c) and location (Fig. 2d). The statistical results show that the performance of real EUS and V-EUS in the diagnosis of benign and malignant tumors in different groups is similar, without significant statistical difference. Several representative examples are shown in Fig. 2e.

### Generalization to multi-center external testing sets

**Fig. 3:**
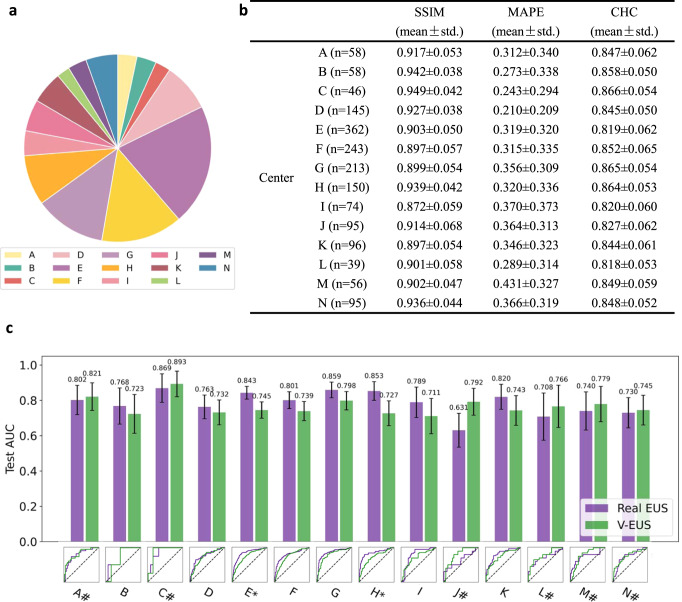
Adaptability to external multi-center external test cohorts. **a** Number of cases comparison of multi-center external test cohort. **b** SSIM, MAPE and CHC comparison in all 14 medical center data. **c** ROC comparison of 14 medical center. * indicates a significant difference (*p* < 0.05, the *p*-value for the center E is 0.0005 and the *p*-value for the center H is 0.0055). # indicates that the AUC of V-EUS is greater than that of real EUS. Error bar indicates 95% confidence intervals of AUC. Source data are provided as a Source Data file.

Due to differences in imaging parameters and clinical settings, US images can vary greatly among different medical centers. It is therefore important to verify that the model trained on the main cohort is robust to different cohorts from other medical centers. We collected 1730 cases from 14 medical centers as external test cohorts to evaluate the generalization performance of the model. The sample distribution of external cohort is shown in Fig. 3a.

We also evaluated the results of V-EUS from two perspectives. For quantitative evaluation, SSIM, MAPE, and CHC were calculated (Fig. 3b). For diagnosing breast cancer, the SR of real EUS and V-EUS were calculated, respectively, and the diagnostic AUCs of each center were analyzed (Fig. 3c). It was found that the diagnostic AUC of V-EUS is not significant different from that of real UES in each centers. These results indicate that our model is capable of generalizing to diverse data sources.

### Tumor depth dependence of diagnostic efficiency

EUS is strongly influenced by imaging attenuation and it was reported to show reduced sensitivity for diagnosing lesions at relatively deep locations^11–13^. With the reconstruction results we found that V-EUS rarely showed artefacts or loss of elastic pseudo-color in deeper tumors. We therefore design experiments to test whether V-EUS is robust to imaging depths. We mixed the main cohort and the multicenter cohort and then all 4231 cases were divided into training and testing datasets according to the depth of tumors. We set 15 mm as the threshold and get 2826 training cases with tumor depth less than 15 mm and 1405 testing cases. We use the AUCs of SR in determining breast malignancy to measure the effectiveness of EUS.

**Fig. 4:**
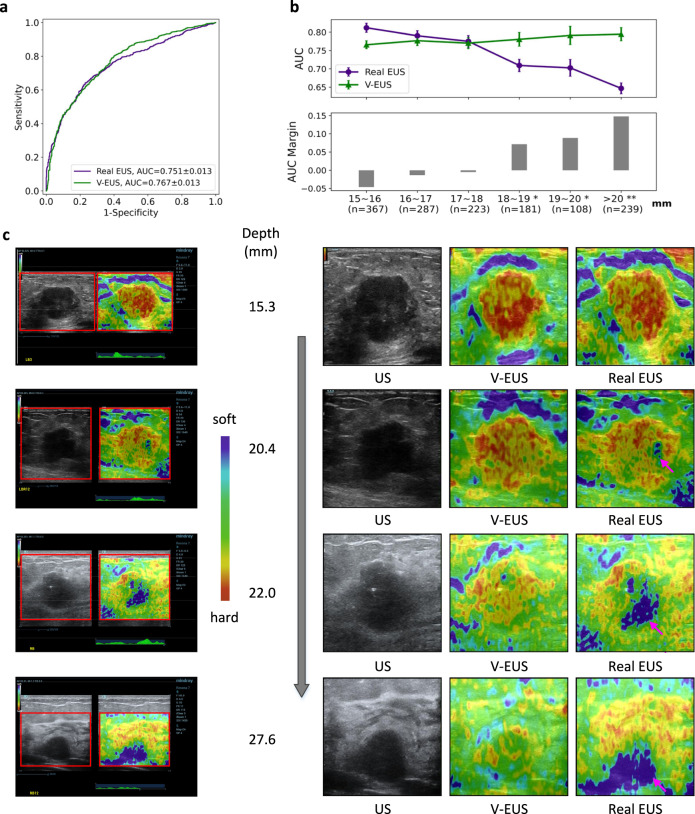
Dependence of V-EUS on tumor depth in diagnosing breast cancer. **a** Comparison of ROCs in determining tumor malignancy on test set when dividing training and test set with 15 mm as the threshold. **b** The diagnostic performance of real EUS and V-EUS varies with the depth of tumor. For the real EUS, the centers of the error bar for each interval are 0.812, 0.790, 0.775, 0.709, 0.702, and 0.647, respectively. For the V-EUS, the centers of the error bar for each interval are 0.766, 0.777, 0.770, 0.781, 0.791 and 0.794 respectively. n indicates the number of cases in the interval. Error bar indicates 95% confidence intervals. (*p < 0.05; **p < 0.01, the *p*-values for the last three intervals are 0.0013, 0.0017, 0.0004 respectively). **c** Examples of typical case results. ROIs were cropped from the US images and displayed on the right together with V-EUS. We observe that for the deep-located tumor, V-EUS not only perform better than real EUS, but also avoid artifacts caused by US signal attenuation. Pink arrows highlight the US imaging at the signal attenuation. Source data are provided as a Source Data file.

The diagnosis AUC of real EUS and V-EUS are 0.751 and 0.767 (Fig. 4a), respectively. The diagnostic performance of V-EUS is not significantly different from that of real EUS. From a more detailed perspective, we statistic the diagnosis AUC for samples of different tumor depth in the test set (Fig. 4b). With increasing tumor depth, the diagnostic performance of V-EUS progressively exceeds that of real EUS.

Representative examples of different tumor types with different tumor depths are shown in Fig. 4c. According to the statistics, we find when the tumor depth is greater than 20 mm, 25.9% (62 of 239) of real EUS exhibit artifacts caused by signal attenuation. It can be seen that V-EUS is more accurate in measuring the hardness of the lesion and can effectively avoid artifacts at the deep-located lesion.

### Blind evaluation on V-EUS

There are two indispensable reasons that motivate us to perform the blind evaluation. One is the gap between human visual perception and computational metrics, and the other is the wide application of the Tsukuba score system (detailed description in Methods) in clinical US examinations. 500 cases were randomly selected from 4231 cases among 15 centers for blind evaluation, which were completed by two radiologists (a senior one with 10 years’ experience and a junior one with 4 years’ experience). To facilitate fair and effective US image evaluation, we released a blind evaluation software for observers (detailed description in “Methods”). During the evaluation, two radiologists were asked to observe a set of real EUS and V-EUS respectively and to give three answers: (1) a corresponding BI-RADS score based on each of the two images, (2) which of the two images was true, and (3) Tsukuba scores for each of the two images based on the Tsukuba scoring system (see “Methods”). The blind evaluation results are summarized in Supplementary Fig. 4.

For the perceptual realism test, if the operator successfully picks out the real one from the two displayed EUS (one is real and the other is virtual), the model is considered to be failed and will score 0. Otherwise, the score will be 1. Therefore, if our model exactly reproduced real EUS, the perceptual score would be 0.5. Interestingly, in the blind test of junior radiologists, the perceptual score is 0.73, indicating our results are deemed more realistic than real EUS. In the blind test of senior US radiologists, the model score is 0.53, which also shows that V-EUS and real EUS are similar in visual authenticity (Supplementary Fig. 4a).

In the experiment on the diagnosis of breast cancer, the Tsukuba scores of real EUS and V-EUS are used as a complement to the BI-RADS scores respectively, thus testing the extent to which they can contribute to the diagnostic performance (the combination method of Tsukuba scores and BI-RADS scores is described in Methods). In the junior radiologists group, the AUC of BI-RADS using the BUS is 0.754, while the AUCs are increased to 0.840 and 0.816 respectively when supplemented with the Tsukuba scoring system based on real EUS and V-EUS. (Supplementary Fig. 4b). In the senior radiologists group, the AUC using BUS is 0.789, while the AUCs are promoted to 0.890 and 0.862 respectively when incorporating with real EUS and V-EUS (Supplementary Fig. 4c).

### Generalization to portable US images

**Fig. 5:**
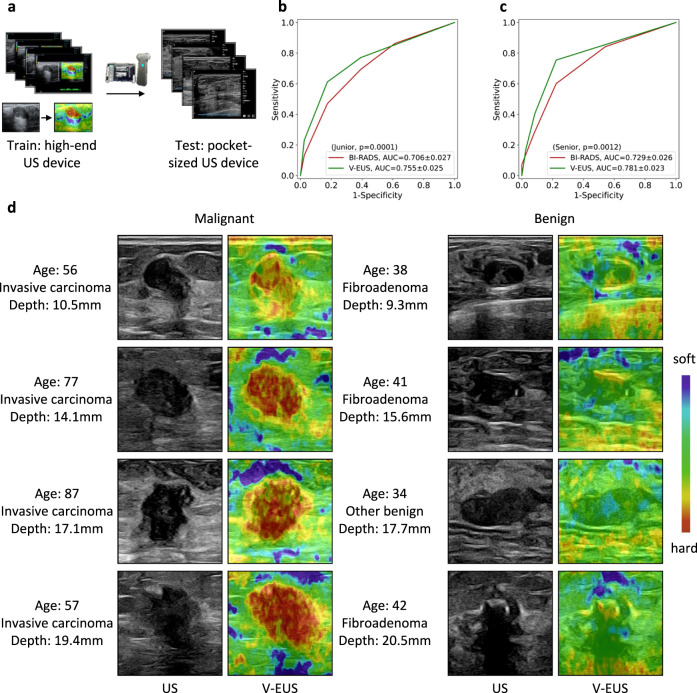
Adaptability to pocket-sized US images. **a** The deep learning model trained on high-quality US images is adapted to low-quality pocket-size US images. **b** ROCs comparison of blind evaluation results of the junior radiologist. **c** ROCs comparison of blind evaluation results of the senior radiologist in diagnosing breast cancer. **d** Examples of typical case results. For lesions with different tumor depth and different benign and malignant types, the model can capture the elastic information effectively. Source data are provided as a Source Data file.

Compared with the US images collected by high-end US devices, the US images collected by pocket-sized US devices have lower resolution, which challenges the generalization ability of the model. Since the pocket-sized US devices cannot perform strain imaging to get the training data, we use US images collected from high-end US devices to train the model and test it with the pocket-sized US images (Fig. 5a). A total of 349 cases with breast tumors were collected by pocket-sized US devices. Similarly, radiologists with different years of experience were involved to perform blind tests. Subjects first performed BI-RADS grading on B-mode US, and then gave the strain scores according to V-EUS. In the junior radiologist group, the AUC of BI-RADS is 0.706, while after using the strain scores of V-EUS, the AUC increases to 0.755 (Fig. 5b). In the senior radiologist group, the AUC of BI-RADS is 0.729, while after using the strain scores of V-EUS, the AUC increases to 0.781 (Fig. 5c). The V-EUS has a significant improvement in determining breast malignancy (*p* = 0.0001 in the junior radiologist group and *p* = 0.0012 in the senior radiologist group). As shown in some examples, we can see that the proposed model can effectively capture the elastic information of the lesion (Fig. 5d).

## Discussion

In this study, we propose a GAN-based model to directly translate US images into V-EUS, which is validated by comprehensive experiments to have good visual consistency and clinical value with real EUS. There are two main considerations in choosing the clinical task of breast cancer diagnosis. First, breast cancer accounts for 30% of malignancies in women, and its incidence continues to increase and result in noteworthy cancer death^14^. Breast cancer screening examinations prior to breast cancer diagnosis can reduce the mortality rate^15^. Second, conventional BUS combined with EUS is becoming the agenda operation and has improved the accuracy of identifying breast malignancies, both in diagnosis and screening^12,16–18^.

Compared with the real EUS acquired by high-end US devices via signal processing, V-EUS avoids artifacts caused by attenuation of ultrasound signals in deep-located tumors. According to the statistical results, we find that among 239 cases with tumor depth greater than 20 mm, there are 62 cases with obvious artifacts caused by ultrasound signal attenuation, accounting for 25.9% of the cases in this group. As a result, the diagnostic performance of real EUS decreased dramatically when the tumor depth is greater than 20 mm. In contrast, the diagnostic performance of V-EUS is hardly affected by tumor depth. In order to provide US radiologists with the superiority of V-EUS in clinical diagnosis, an envisage is that if the tumor depth is greater than 20 mm, radiologists use the results of V-EUS, otherwise they use the real EUS provided by US devices. Applying this idea to our retrospective study, we observe a significant improvement in breast cancer diagnosis (*p* < 0.05). It is worth nothing that our study is based on clinical data of Asian patients, and the results may be more pronounced for European, American and African patients, who tend to have deeper breast tumor than Asian patients.

In addition to providing assistance for high-end US devices, V-EUS has a more profound impact on pocket-sized US devices that cannot perform EUS imaging. The current dilemma is that although the market share of pocketed-sized US devices is increasing in recent years due to its high flexibility and low cost, it is currently unable to perform EUS imaging for the limitation of imaging hardware. In resource-limited areas, portable US scanner, rather than standard high-end US scanner, could serve as a primary detection modality for early breast cancer detection because of its portability and low cost^19^. However, due to cost and size limitations, the function of portable US scanner is limited, for example, it does not have the function of elastography, which can improve the accuracy of breast cancer screening as mentioned above. V-EUS provides a solution with almost hardware cost free for pocket-sized US devices. In this study, we train the deep learning model with paired BUS and EUS images acquired from high-end US devices and test the model with BUS acquired from pocket-sized US devices without any fine-tuning or domain adaption. The diagnostic results and examples shown in Fig. 5 demonstrate that V-EUS has a great potential to empower the pocket-sized US devices. To better exploit V-EUS, we also explore the performance of the model after domain adaption. We first standardized the US images acquired from pocket-sized US devices through a CycleGAN model and then use the standardized US images to generate V-EUS^20,21^. Results are shown in Supplementary Fig. 5. The results illustrate that by standardizing the US images, more realistic V-EUS images can be generated. Similarly, blind evaluation was performed and the results showed that the virtual EUS generated from BUS after domain adaption did not significantly improve the diagnostic performance of breast cancer (*p* > 0.05), which indicates that our model has the ability to synthesize V-EUS with clinical value from original pocket-sized US.

Although we have demonstrated that V-EUS performs well in the clinical task of breast cancer diagnosis, there are many aspects of future work that can be extended. From the perspective of clinical tasks, the effectiveness of V-EUS in the diagnosis of other breast diseases and the imaging of other organs, such as thyroid and liver, still need to be proved. In fact, the deep learning model proposed in this work is very convenient to transfer to other US image synthesis tasks. Using the shear wave elastography (SWE) images as training labels, the model can establish a mapping from B-mode US images to SWE images. If the clinical effectiveness of the synthesized SWE images can be proved, it will have a profound impact on the development and clinical application of US devices.

In conclusion, we present a deep learning framework for synthesizing V-EUS through BUS, and validate the clinical value of V-EUS in diagnosing breast cancer through comprehensive experiments. V-EUS can not only provide high-end US devices with accurate diagnostic results in examining deep located tumors, but more importantly, endow the pocket-sized US devices with the capability of performing EUS imaging.

## Methods

### Data collection and pre-processing

This study, carried out from August 2016 to March 2021, was approved by the Ruijin Hospital Ethics Committee, Shanghai Jiao Tong University School of Medicine, and written informed consent to participate were acquired before examinations. All patients in the 15 centers underwent core needle biopsy or surgery after conventional US and elastography examination, and thus the histopathological findings were obtained for all breast lesions. The high-end US instrument used was the Resona 7 ultrasound system (Mindray Medical International, Shenzhen, China) equipped with L11-3 high-frequency probe, and the pocket-sized US device used was the Stork diagnostic ultrasound system (Stork Healthcare Co., Ltd. Chengdu, China) with L12-4 high-frequency probe.

Prior to data collection, all participating US radiologists from the 15 hospitals with at least 3 years of experience in breast elastography have received standard training in both conventional US and elastography examination of breast. After quality control by radiologists with more than 10 years of experiences on breast elastography, the ultrasound data consisting of 4231 paired BUS and EUS from high-end instruments and 349 B-mode US images from pocket-sized devices.

In clinical setting, BUS and EUS of breast lesions are completely aligned, and the target imaging area is determined by the experienced radiologists. A typical US image is shown in Supplementary Fig. 1. The proposed model aims to learn a statistical transformation between BUS and EUS. In order to reduce the influence of irrelevant information in the US image, we extract the region of interest (ROI) marked by the radiologists, and use the fully registered ROIs of BUS and EUS as the input and the target of the model. For pocked-size US images, tumor ROIs are also marked by radiologists and sent to the model for inference.

### Deep neural network architecture, training and validation

#### Overall architecture of the model

As illustrated in Fig. 1, the GAN based deep learning model consists of a generator and a discriminator, both of which are in a dynamic game process during training^22^.

#### Generator

Under the paradigm of the GAN model, the architecture of the generator follows the design of U-net^23^, as shown in Fig. 1. It is pretty suitable to use U-net structure for this study. In addition to learning the overall mapping relationship between inputs and outputs, the encoder-decoder structure of the model is helpful to learn semantic information at different scale. The skip connection between encoder and decoder ensures that the decoder can integrate more low-level features which is essential for enriching the details of EUS image^24^.

After data preprocess, the input BUS with a size of 256*256 were feed into the generator. In the encoder, it contains an input layer and 6 convolutional blocks. Each convolutional block is composed of a ReLU layer, a convolutional layer and a batch-normalization layer^25^. Between each convolutional block, we used convolution with step size of 2 instead of down-sampling, which may decrease the information loss^26^. The output channels of each convolutional block in encoder was set to 64, 128, 256, 512, 512, 512, 512.In the decoder, it contains 6 convolutional blocks and an output layer. Different from the convolutional blocks in the encoder, the convolution operation in decoder is replaced by the deconvolution operation, which reconstructs the feature map back to the input image size. The input channels of each convolutional blocks in decoder was set to 512, 1024, 1024, 1024, 512, 256, 128. The last layer is a deconvolution operation followed by a Tanh activation layer, which mapping 128 channels feature maps into 3 channels EUS.

#### Discriminator

The discriminator, as shown in Fig. 1, receives 4-channel composite image (concatenating 1-channel BUS and 3-channel EUS) as input. This is a paradigm of conditional GAN, which aims to expose the discriminator to more prior knowledge^27^. The 4 channels composite image is then feed into a convolutional layer followed by 4 convolutional blocks and an output layer. Each convolutional block is composed of a convolution layer, a batch-norm layer and a Leaky-ReLU activation layer. The output channels of each convolution layer in discriminator were set to 64, 128, 256, 512, 512 and 1. The local connection characteristic of convolution operation makes patch retain the spatial information of the input image, so the discriminator effectively models the input image as a Markov random field, which is crucial for high-frequencies reconstruction^27,28^.

#### Tumor discriminator

The aim of elasticity reconstruction is to accurately evaluate the degree of elasticity of the tumor. Therefore, in addition to the global discriminator used to classify whether the input image is real or fake, we further designed a local discriminator to determine whether the tumor area is real or fake. Because the color distribution of the tumor region is different from the normal tissue on EUS, the local discriminator, by taking tumor area as input, can effectively distinguish between tumor tissue and normal tissue, thus improving the realism of the elastic reconstruction of the tumor region. (see example in Supplementary Fig. 6). The results of the ablation test show that tumor discriminator has improved the quality of V-EUS (see results in Supplementary Table 1).

#### Color rebalancing

A remarkable characteristic of EUS is the simple color distribution, with blue and red dominating most of the color distribution. As shown by the empirical distribution of pixels in lab space shown in Supplementary Fig. 7, the output of the model has a tendency to be dominated by a large number of color types if distribution differences are not taken into account, which may reduce the realism of the virtual strain images. To accommodate this, we proposed a color-rebalancing coefficient to reweight L1 loss during training based on the color rarity. We statistic and calculate the color distribution in lab color space. Compared with RGB space, lab space is more in line with the visual perceptual and convenient for calculation^29^. The factor $\gamma \in {{\mathbb{R}}}^{Q}$ is defined by Eq. (1):

1 $${\gamma }_{p}={(\alpha \widetilde{P}+(1-\alpha )/Q)}^{-1}$$

where $\widetilde{P}$ is the empirical distribution of pixel $p$, ${Q}$ is the number of quantized *ab* space, so $1/Q$ is a uniform distribution and we mixed the prior distribution and uniform distribution with weight $\alpha \in [{{{{\mathrm{0,1}}}}}]$. In our experiment, $\alpha=0.8$ works well. The results of the ablation test show that it has improved the quality of V-EUS images (see results in Supplementary Table 1).

#### The calculation methods of SSIM, MAPE, CHC

SSIM, MAPE and CHC are metrics that commonly used to evaluate the similarity between two images. The calculation formulas are listed as below:

The ${{{{{\rm{SSIM}}}}}}$ is a metric to measure the similarity between two images, as defined in (2):

2 $${{{{{\rm{SSIM}}}}}}=\frac{(2{\mu }_{{real}}{\mu }_{{virtual}}+{C}_{1})(2{\sigma }_{r{eal},{virtual}}+{C}_{2})}{({\mu }_{{real}}^{2}+{\mu }_{{virtual}}^{2}+{C}_{1})({\sigma }_{{real}}^{2}+{\sigma }_{{virtual}}^{2}+{C}_{2})}$$

where ${\mu }_{{real}}$ and ${\mu }_{{virtual}}$ are the average of real EUS and V-EUS, respectively. ${\sigma }_{{real}}$ and ${\sigma }_{{virtual}}$ are the variance of real EUS and V-EUS, respectively. ${\sigma }_{{real},{virtual}}$ is the covariance of real EUS and V-EUS. ${C}_{1}$ and ${C}_{2}$ are constants.

The MAPE denotes percentage of the mean absolute error between SR of real EUS and V-EUS, as defined in (3):

3 $${{{{{\rm{MAPE}}}}}}=\frac{1}{m}\mathop{\sum }\limits_{i=1}^{m}\frac{\left|{p}_{i}^{{real}}-{p}_{i}^{{virtual}}\right|}{{p}_{i}^{{real}}}$$

where ${p}_{i}^{{real}}$ and ${p}_{i}^{{virtual}}$ represents the strain scores of real EUS and V-EUS, respectively.

The CHC is a metrics to measure the histogram correlation (HC) between two images in hue and saturation color space. The HC is defined in (4):

4 $${{{{{\rm{HC}}}}}}=1-\sqrt{1-\frac{\sum \sqrt{{{Cnt}}_{{real}}\cdot {{Cnt}}_{{virtual}}}}{\sqrt{\sum {{Cnt}}_{{real}}\cdot \sum {{Cnt}}_{{virtual}}}}}$$

where the ${{Cnt}}_{{real}}$ and ${{Cnt}}_{{virtual}}$ are vectors containing the count of every bin in the histogram of real EUS and V-EUS respectively. Therefore, CHC is the mean of HC in hue and saturation color space.

#### Decoding pseudo color of EUS via color bar and strain ratio calculation

A typical US image collected from high-end US instrument (Resona 7 ultrasound system, Mindray Medical International, Shenzhen, China) is shown in Supplementary Fig. 1. The pseudo color which depicts tissue elasticity is overlaid on BUS to form EUS, as shown in the right of Supplementary Fig. 1.

As shown by the color bar in the upper left of Supplementary Fig. 1, the red in EUS indicates that the tissue is hard, and the blue indicates that the tissue is soft. In order to qualify the difference in tissue elasticity represented by different colors in EUS, we use the color bar to decode the colors in EUS. Specifically, we first subtract BUS from EUS to obtain the pure color. Then, we decode the pure color image by using the color bar^30^. The elasticity modulus value is encoded with 256 pseudo-color levels from blue to red. Each pixel in pure color image is compared with all the color in color bar and the modules value with the smallest distance is taken as the elasticity value at this pixel. The distance ${{{{{\rm{d}}}}}}$ between the pixel and the color is defined in (5):

5 $${{{{{\rm{d}}}}}} \, \left({{{{{\rm{c}}}}}}\right)=\sqrt{{({R}_{{pixel}}-{R}_{c})}^{2}+{({G}_{{pixel}}-{G}_{c})}^{2}+{({B}_{{pixel}}-{B}_{c})}^{2}}$$

where $R$, ${G}$, ${B}$ is three color channel and the subscripts ${pixel}$ and ${{{{{\rm{c}}}}}}$ represent the pixel in pure strain image and pixel in color bar. In our experiment, the color bar is divided in to 256 color classes from blue to red, so the range of ${{{{{\rm{c}}}}}}$ is 1 to 256. This process is shown in Supplementary Fig. 2.

To calculate the SR, we also select a reference area with a size of 25*25 pixels in the pure strain image, decode it and average the modules value to obtain the elastic value of the reference region. According to the definition of SR, we divide the elastic value of tumor region and reference region to obtain the SR.

#### Blind evaluation

As shown in Supplementary Fig. 3, the blind evaluation software mainly composed of 3 modules, including real EUS and V-EUS judgement, BUS BI-RADS grading and EUS 5-point scoring. The process of blind evaluation is as follows:

1. Participant chose their working years (senior or junior) in the menu, and then select the folder to be evaluated.
2. The US image pair being evaluated is displayed in the image display area at the bottom of the software interface. The display position of BUS is fixed and always displayed on the left side of the image display area. Real EUS and V-EUS are randomly displayed on the right side of the BUS, and their positions are marked as image 1 and image 2 respectively. Participant is requested to give the BI-RADS score of BUS, the Tsukuba score of image 1 and image 2, and pick out the real EUS from image 1 and image 2.
3. The status window on the right side of the software interface outputs the current evaluation status. Click the ‘Next’ button to evaluate the next pair of US images.

#### The Tsukuba scoring system

The EUS is scored by sonographer based on 5-point scoring system^17^. The detailed scoring rules are as follows: 1 = even strain over the entire lesion; 2 = strain in most of the lesion; 3 = strain at the periphery of the lesion with sparing in the center of a lesion; 4 = no strain in the entire lesion; 5 = no strain in the entire lesion or the surrounding area. The Tsukuba score and BI-RADS are combined in the following way: downgrade BI-RADS category in lesions with the Tsukuba score 1–3, while upgrade BI-RADS category in lesions with the Tsukuba score 4–5.

#### Loss function

Ultimately, the loss function of generator and discriminator is defined by Eq. (6):

6 $${{{{{{\mathcal{L}}}}}}}_{{generator}}	={{{{{\rm{\lambda }}}}}}*\gamma*{{{{{{\mathcal{L}}}}}}}_{L1}\left({y}_{{real}},\,{y}_{{virtual}}\right)+[{{{{{{\mathcal{L}}}}}}}_{{Cross}-{entropy}}\left(1,\,D\left(x,\,{y}_{{virtual}}\right)\right)+{{{{{{\mathcal{L}}}}}}}_{{Cross}-{entropy}}\left(1,\,D\left({x}_{{tumor}},\,{y}_{{virtual}}^{{tumor}}\right)\right)]\\ {{{{{{\mathcal{L}}}}}}}_{{discriminator}}^{{global}}	={{{{{{\mathcal{L}}}}}}}_{{Cross}-{entropy}}\left(1,\,D\left(x,\,{y}_{{real}}\right)\right)+{{{{{{\mathcal{L}}}}}}}_{{Cross}-{entropy}}\left(0,\,D\left(x,\,{y}_{{real}}\right)\right)\\ {{{{{{\mathcal{L}}}}}}}_{{discriminator}}^{{tumor}}	={{{{{{\mathcal{L}}}}}}}_{{Cr}{oss}-{entropy}}\left(1,\,D\left({x}_{{tumor}},\,{y}_{{real}}^{{tumor}}\right)\right)+{{{{{{\mathcal{L}}}}}}}_{{Cross}-{entropy}}\left(0,\,D\left({x}_{{tumor}},\,{y}_{{virtual}}^{{tumor}}\right)\right)\,{Loss}={{{{{{\mathcal{L}}}}}}}_{{generator}}+0.5*{{{{{{\mathcal{L}}}}}}}_{{discriminator}}^{{global}}+{0.5*{{{{{\mathcal{L}}}}}}}_{{discriminator}}^{{tumor}}$$

where $x$ denotes the input B-mode image, ${y}_{{virtual}}$ and ${y}_{{real}}$ refer to the V-EUS image and the real EUS image respectively, ${y}_{{virtual}}^{{tumor}}$ and ${y}_{{real}}^{{tumor}}$ refer to the tumor area cropped from ${y}_{{virtual}}$ and ${y}_{{real}}$ respectively. The factor ${{{{{\rm{\lambda }}}}}}$ is empirically set to 100 to accommodate MAE loss and Cross-entropy loss from discriminator.

#### Training parameters

The generator and discriminator of the model update the parameters alternately in the training process. The max training epoch is set to 200 with a batch size of 1 empirically. The model parameters are updated via the Adam optimizer with a learning rate of 2*10-4, which linearly decay to zero staring from epoch 100. All convolution kernels were set to 4*4 and were randomly initialized by using a normal distribution. In order to improve the generalization ability considering the limited medical data, we apply a differentiable data augmentation algorithm during training^31^. In addition, we also augmented the training data by applying random cropping and random flipping. Once the model training is finished, the inference only involves the use of generator, so no additional annotation is required.

#### Implementation details

The deep neural network was implemented using Python version 3.6.2 and the model was built based on Pytorch version 1.7.1. Some other python libraries used in this project were os, time, the Python Imaging Library (PIL), visdom, argparse, skimage and numpy. We implemented this software on a 24-core CPUs workstation with 3 3090 Nvidia GPUs.

### Statistical analysis

Descriptive statistics were summarized as mean ± SD or median. The diagnostic performances of the real EUS and V-EUS for differentiating breast cancer were expressed as AUC values with 95% confidence intervals (CIs) were calculated. Comparisons between AUCs were made by using Delong test. *P* < 0.05 was considered to have a significant difference. The statistical analyses were performed using MedCalc software, version 11.2 [MedCalc Software, Ostend, Belgium].

### Reporting summary

Further information on research design is available in the Nature Portfolio Reporting Summary linked to this article.

## Supplementary information

Supplementary Information

Reporting Summary

## Data Availability

Source Data are provided with this paper. All data associated with this study are available from the department of ultrasound in Ruijin Hospital. Requests for academic use of in-house raw data can be addressed to the corresponding author. All requests will be promptly reviewed to determine whether the request is subject to any intellectual property or patient-confidentiality obligations, will be processed in concordance with institutional and departmental guidelines and will require a material transfer agreement. Source data are provided with this paper.

## Source data

Source Data

## Code availability

All code was implemented in Python using PyTorch as the primary deep-learning library. All source codes are available at https://github.com/yyyzzzhao/VEUS.
