# Supplementary material for: Virtual elastography ultrasound via generative adversarial network for breast cancer diagnosis
Source: Nat Commun. 2023 Feb 11;14:788. doi: 10.1038/s41467-023-36102-1 (PMC9922312; doi:10.1038/s41467-023-36102-1)
Supplement: Supplementary file 1 — Supplementary Information [file 41467_2023_36102_MOESM1_ESM.pdf]

Title: Virtual elastography ultrasound via generative adversarial network for breast cancer diagnosis

Supplementary Figures

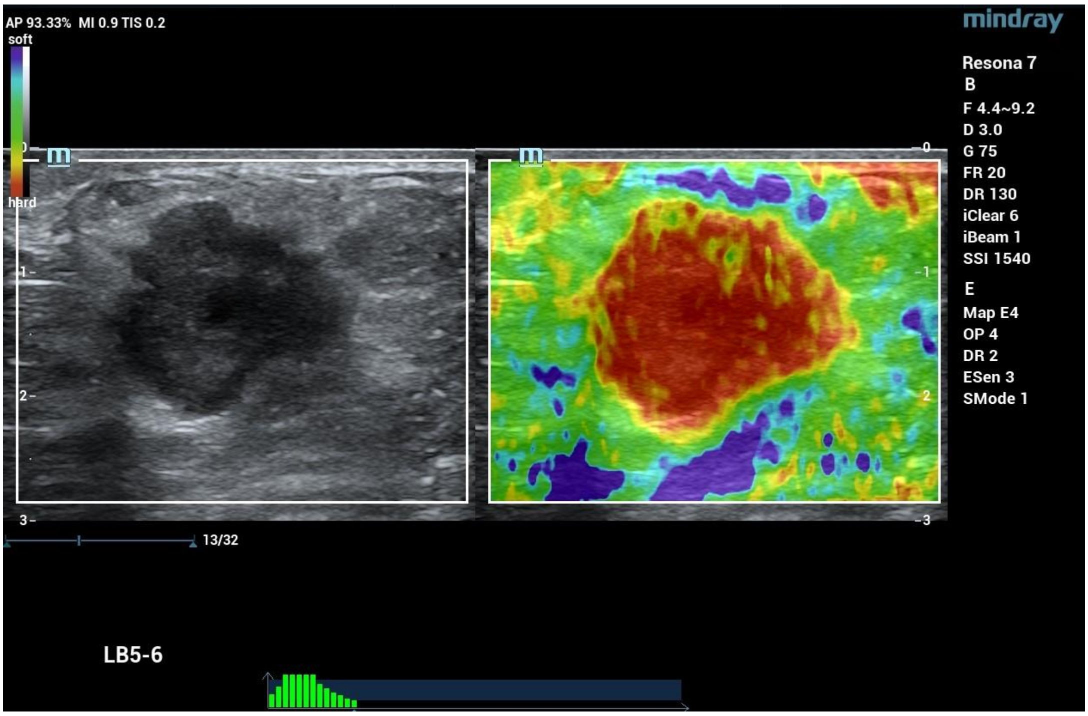

Supplementary Fig. 1. An example of US image collected from high-end US device. In this figure, BUS and EUS are shown in the white boxes on the left and right sides respectively.

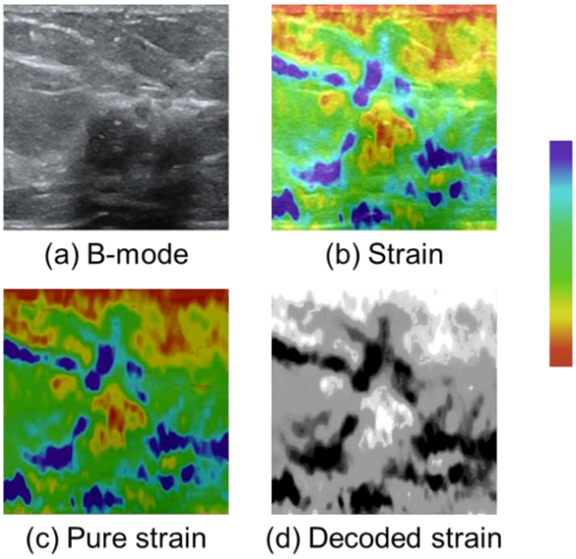

Supplementary Fig. 2. Pseudo color image decoding process. **a** the B-mode US image; **b** the original strain overlay on the B-mode US image by pseudo-color. **c** pure strain image obtained by subtracting the B-mode US image from the strain image. **d** gray-scale strain image reconstructed by decoding pure strain image.

7

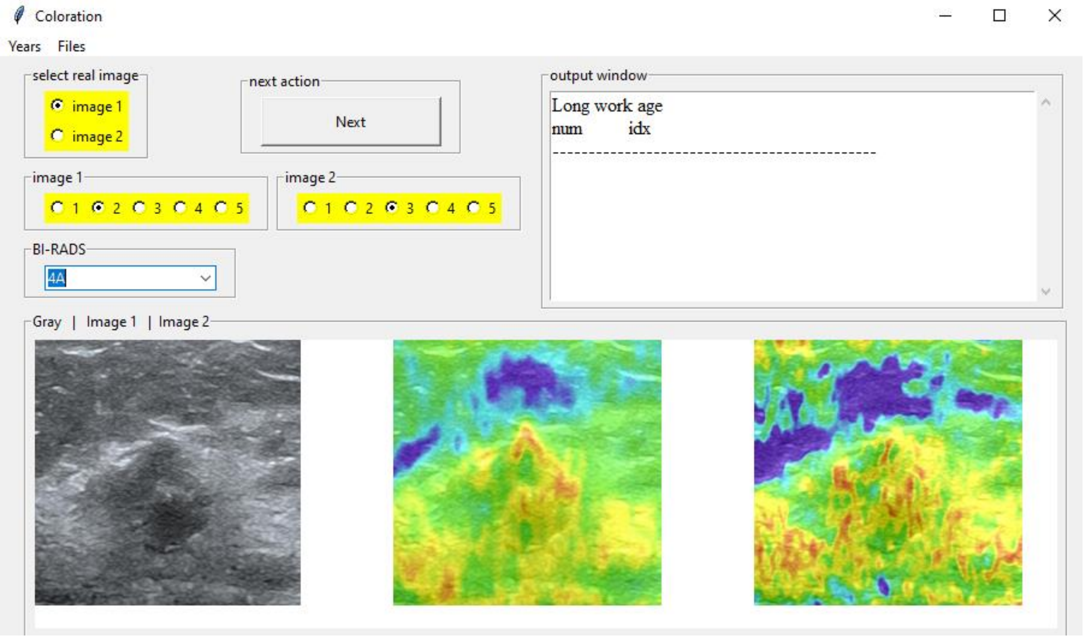

Supplementary Fig. 3. Software interface for blind evaluation.

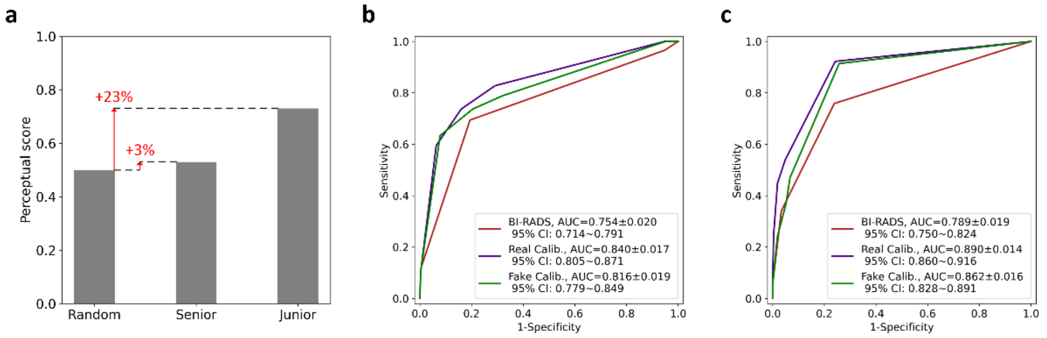

Supplementary Fig. 4. Blind evaluation results of V-EUS and real EUS. a. Perceptual score comparison of blind evaluation results of the junior radiologist and senior radiologist with random group. b. ROCs comparison of blind evaluation results of the junior radiologist in diagnosing breast cancer using BI-RADS, real EUS combined with BI-RADS, V-EUS combined with BI-RADS. c. ROCs comparison of blind evaluation results of the senior radiologist in diagnosing breast cancer using BI-RADS, real EUS combined with BI-RADS, V-EUS combined with BI-RADS.

9

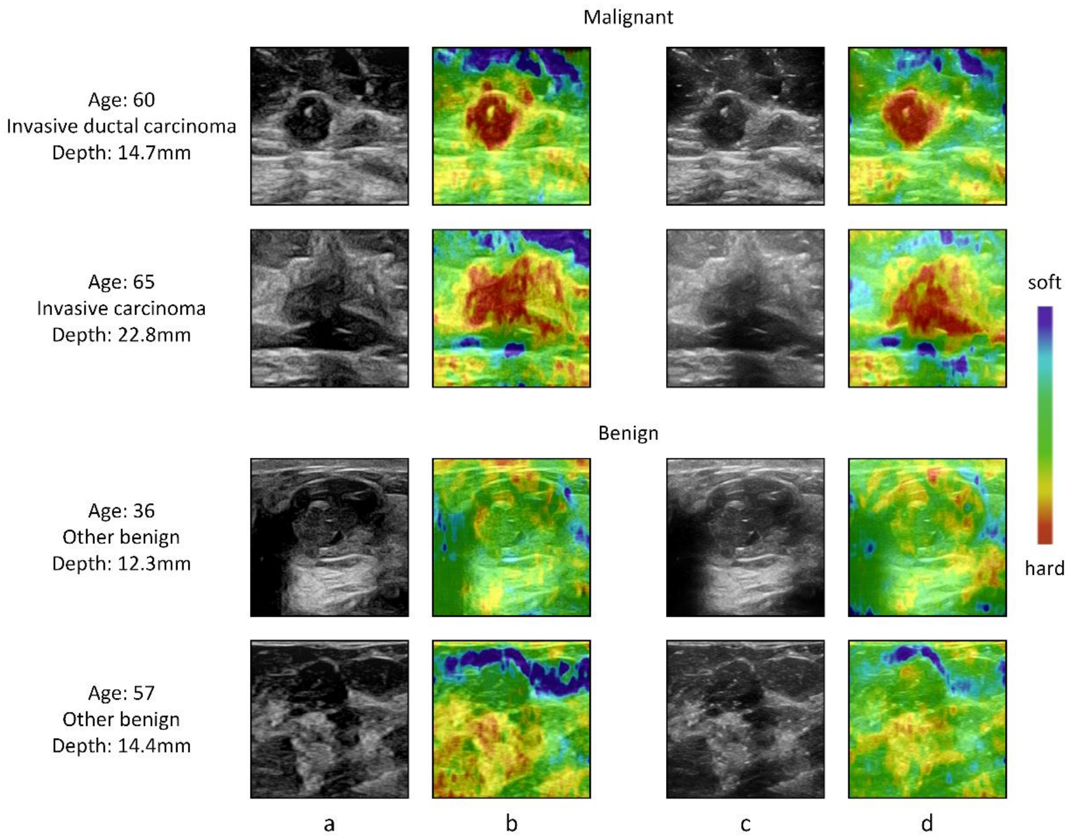

Supplementary Fig. 5. Comparison of results before and after standardization of low-quality US. a. low-quality US. b. V-EUS generated from low-quality US. c. Standardized low-quality US. d. V-EUS generated from standardized low-quality US.

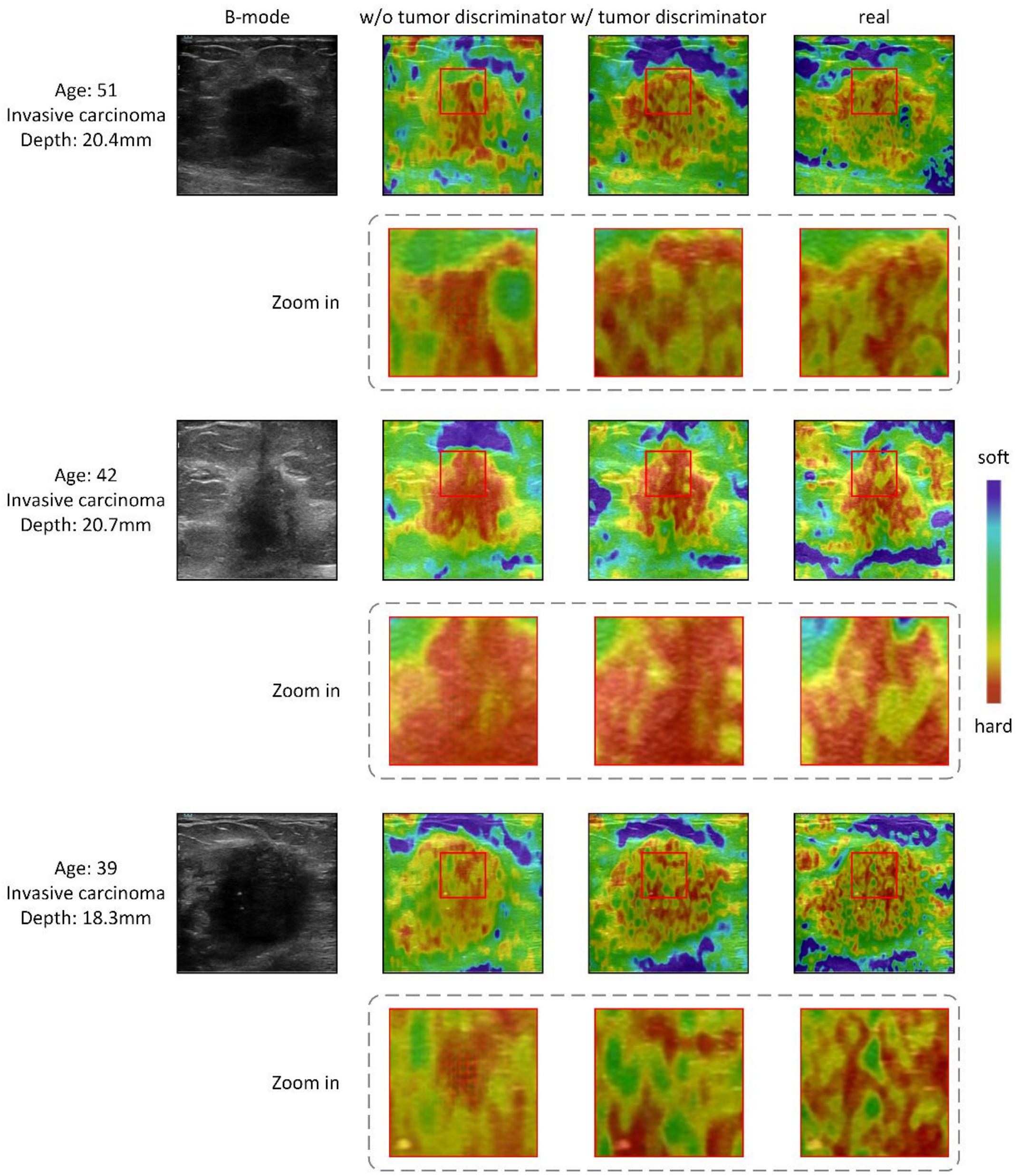

Supplementary Fig. 6. Examples of V-EUS generated by model with or without tumor discriminator. For each example, we show BUS, real EUS and two V-EUS images generated by model with or without tumor discriminator. The zoomed area (marked by red box) shows that the V-EUS generated by the model with tumor discriminator can effectively avoid the ‘checkerboard’ artifacts.

11

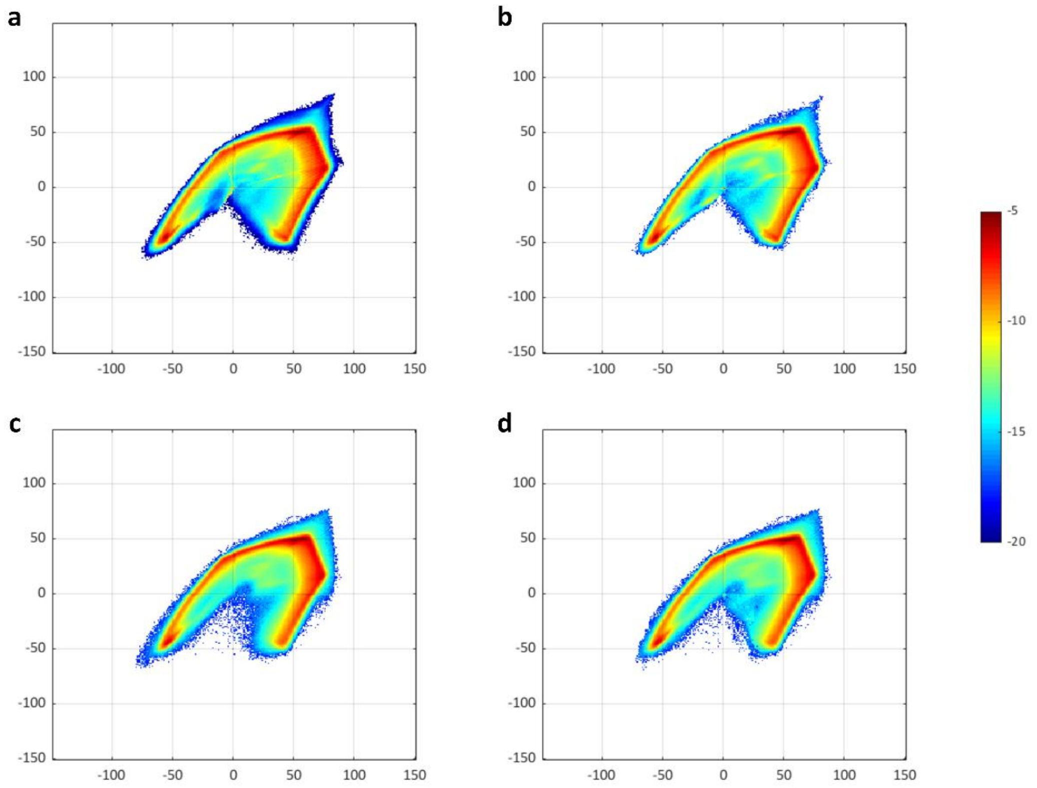

Supplementary Fig. 7. Comparison of the empirical distribution of *ab* values, shown in log scale. **a** The empirical distribution of *ab* values in train set. **b** The empirical distribution of *ab* values of real EUS in test set. **c** The empirical distribution of *ab* values of V-EUS in test set without using prior boost module. **d** The empirical distribution of *ab* values of V-EUS in test set using prior boost module. When the prior boost module is not added, the *ab* value distribution of V-EUS is quite different from that of real EUS. By adding the prior boost module to the model, the *ab* value distribution of V-EUS is consistent with that of real EUS.

Supplementary Tables

Supplementary Table 1. Comparison and ablation experiment results

	Tumor enhance	Prior boost	SSIM	PSNR	MAE
[1]	-	-	0.868±0.072	20.481±3.219	8.929±4.128
[2]	-	-	0.876±0.062	22.015±3.026	6.383±3.678
Exp. 1	-	-	0.896±0.062	22.088±3.068	6.282±3.713
Exp. 2	√	-	0.897±0.062	22.364±3.100	6.058±3.604
Exp. 3	-	√	0.887±0.055	22.167±3.239	5.980±3.530
Exp. 4	√	√	**0.903±0.059**	**22.464±3.090**	**5.908±3.495**

Supplementary References

1 Wildeboer, R. R. *et al.* Synthetic Elastography Using B-Mode Ultrasound Through a Deep Fully Convolutional Neural Network. *Ieee T Ultrason Ferr* **67**, 2640-2648 (2020).

2 Zhang, Q. J. *et al.* AUE-Net: Automated Generation of Ultrasound Elastography Using Generative Adversarial Network. *Diagnostics* **12** (2022).
