# Supplementary material for: Virtual elastography ultrasound via generative adversarial network for breast cancer diagnosis
Source: Nat Commun. 2023 Feb 11;14:788. doi: 10.1038/s41467-023-36102-1 (PMC9922312; doi:10.1038/s41467-023-36102-1)
Supplement: Supplementary file 2 — Reporting Summary [file 41467_2023_36102_MOESM2_ESM.pdf]

nature portfolio

Corresponding author(s): JinHua Yu, JianQiao Zhou

Last updated by author(s): Jan 17, 2023

# Reporting Summary

Nature Portfolio wishes to improve the reproducibility of the work that we publish. This form provides structure for consistency and transparency in reporting. For further information on Nature Portfolio policies, see our Editorial Policies and the Editorial Policy Checklist.

## Statistics

For all statistical analyses, confirm that the following items are present in the figure legend, table legend, main text, or Methods section.

| n/a | Confirmed | |
|---|---|---|
| ☐ | ☒ | The exact sample size (*n*) for each experimental group/condition, given as a discrete number and unit of measurement |
| ☒ | ☐ | A statement on whether measurements were taken from distinct samples or whether the same sample was measured repeatedly |
| ☐ | ☒ | The statistical test(s) used AND whether they are one- or two-sided<br>*Only common tests should be described solely by name; describe more complex techniques in the Methods section.* |
| ☒ | ☐ | A description of all covariates tested |
| ☒ | ☐ | A description of any assumptions or corrections, such as tests of normality and adjustment for multiple comparisons |
| ☐ | ☒ | A full description of the statistical parameters including central tendency (e.g. means) or other basic estimates (e.g. regression coefficient) AND variation (e.g. standard deviation) or associated estimates of uncertainty (e.g. confidence intervals) |
| ☐ | ☒ | For null hypothesis testing, the test statistic (e.g. *F*, *t*, *r*) with confidence intervals, effect sizes, degrees of freedom and *P* value noted<br>*Give P values as exact values whenever suitable.* |
| ☒ | ☐ | For Bayesian analysis, information on the choice of priors and Markov chain Monte Carlo settings |
| ☒ | ☐ | For hierarchical and complex designs, identification of the appropriate level for tests and full reporting of outcomes |
| ☒ | ☐ | Estimates of effect sizes (e.g. Cohen's *d*, Pearson's *r*), indicating how they were calculated |

*Our web collection on statistics for biologists contains articles on many of the points above.*

## Software and code

Policy information about availability of computer code

| | |
|---|---|
| Data collection | All patients in the 15 centers underwent core needle biopsy or surgery after conventional US and elastography examination, and thus the histopathological findings were obtained for all breast lesions. The high-end US instrument used was the Resona 7 ultrasound system (Mindray Medical International, Shenzhen, China) equipped with L11-3 high-frequency probe, and the pocket-sized US device used was the Stork diagnostic ultrasound system (Stork Healthcare Co., Ltd. Chengdu, China) with L12-4 high-frequency probe. |
| Data analysis | The deep neural network was implemented using Python version 3.6.2 and the model was built based on Pytorch version 1.7.1. Some other python libraries used in this project were os, time, the Python Imaging Library (PIL, version 8.4.0), visdom (version 0.1.8.8), argparse (version 1.1), skimage (version 0.17.2) and numpy (version 1.19.5). The statistical analyses were performed using MedCalc software, version 11.2 [MedCalc Software, Ostend, Belgium]. The implementation of the model is publicly available at https://github.com/yyyzzzhao/VEUS. |

For manuscripts utilizing custom algorithms or software that are central to the research but not yet described in published literature, software must be made available to editors and reviewers. We strongly encourage code deposition in a community repository (e.g. GitHub). See the Nature Portfolio guidelines for submitting code & software for further information.

## Data

Policy information about availability of data

All manuscripts must include a data availability statement. This statement should provide the following information, where applicable:

- Accession codes, unique identifiers, or web links for publicly available datasets
- A description of any restrictions on data availability
- For clinical datasets or third party data, please ensure that the statement adheres to our policy

Source Data are provided with this paper. All data associated with this study are available from the department of ultrasound in Ruijin Hospital. Requests for academic use of in-house raw data can be addressed to the corresponding author. All requests will be promptly reviewed to determine whether the request is subject to any intellectual property or patient-confidentiality obligations, will be processed in concordance with institutional and departmental guidelines and will require a material transfer agreement.

## Human research participants

Policy information about studies involving human research participants and Sex and Gender in Research.

| | |
|---|---|
| Reporting on sex and gender | Breast tumors occur almost exclusively in women. In our dataset, we included data on 4578 women and 2 men, so our model was validated on women, but not on men due to lack of validation data. |
| Population characteristics | Baseline characteristics of cases are reported in Table 1. Briefly, patients were aged 48±14 on average, with only 2 men cases, and the most common of the malignant tumors being invasive ductal carcinoma and the most common of the benign tumors being fibroadenoma. |
| Recruitment | All consecutive adult cases in the time period (2016-2021) were used, excluding those without pathological confirmation or without complete modality, where there is no self-selection bias or other bias. |
| Ethics oversight | This study was approved by the Ruijin Hospital Ethics Committee, Shanghai Jiao Tong University School of Medicine. |

Note that full information on the approval of the study protocol must also be provided in the manuscript.

# Field-specific reporting

Please select the one below that is the best fit for your research. If you are not sure, read the appropriate sections before making your selection.

☒ Life sciences ☐ Behavioural & social sciences ☐ Ecological, evolutionary & environmental sciences

For a reference copy of the document with all sections, see nature.com/documents/nr-reporting-summary-flat.pdf

# Life sciences study design

All studies must disclose on these points even when the disclosure is negative.

| | |
|---|---|
| Sample size | The study was based on dataset with 4580 cases, including a main cohort with 2501 cases, an external cohort with 1730 cases and a portable dataset with 349 cases. We used all available data for model training and testing. |
| Data exclusions | We excluded some cases with no pathological confirmation or incomplete modality. |
| Replication | We made the experimental code public, and experimental data is available under reasonable application, so we confirm that the experimental results can be reproduced. We confirm that all attempts at replication were successful. |
| Randomization | In the main cohort, we random selected 500 cases as testing set and the rest 2001 cases as training set. In the multi-center cohort and portable cohort, we did not partition the data. |
| Blinding | The investigators were blind to group allocation during data collection and analysis. |

# Reporting for specific materials, systems and methods

We require information from authors about some types of materials, experimental systems and methods used in many studies. Here, indicate whether each material, system or method listed is relevant to your study. If you are not sure if a list item applies to your research, read the appropriate section before selecting a response.

## Materials & experimental systems

| n/a | Involved in the study | |
|---|---|---|
| ☒ | ☐ | Antibodies |
| ☒ | ☐ | Eukaryotic cell lines |
| ☒ | ☐ | Palaeontology and archaeology |
| ☒ | ☐ | Animals and other organisms |
| ☒ | ☐ | Clinical data |
| ☒ | ☐ | Dual use research of concern |

## Methods

| n/a | Involved in the study | |
|---|---|---|
| ☒ | ☐ | ChIP-seq |
| ☒ | ☐ | Flow cytometry |
| ☒ | ☐ | MRI-based neuroimaging |
